# Supplementary material for: Insight into dynamic and steady-state active sites for nitrogen activation to ammonia by cobalt-based catalyst
Source: Nat Commun. 2020 Jan 31;11:653. doi: 10.1038/s41467-020-14287-z (PMC6994663; doi:10.1038/s41467-020-14287-z)
Supplement: Supplementary file 1 — Supplementary Information [file 41467_2020_14287_MOESM1_ESM.pdf]

Wang et al.

Insight into dynamic and steady-state active sites for nitrogen activation to ammonia by cobalt-based catalyst

Supplementary Information

Supplementary Methods

$^{15}N_2$ isotopic exchange over Co-N-C

The number of N atoms that have undergone isotopic exchanged (N_e) in the case of Co-N-C was calculated as described elsewhere[1,2].

$$\alpha_g = \frac{P_{30} + \frac{1}{2}P_{29}}{P_{30} + P_{29} + P_{28}} \qquad [1]$$

where P_{30}, P_{29} and P_{28} represent the partial pressures of $^{15}N_2$, $^{14}N^{15}N$ and $^{14}N_2$, respectively.

$$N_e = N_g\,(1 - \alpha_g) \qquad [2]$$

where N_g is the number of ^{15}N atoms in the outlet gas.

Finally, the number of exchangeable N atom was calculated when the exchange between gas phase $^{15}N_2$ and N atom in Co-N-C is close to equilibrium according to the following equation:

$$N_s = \frac{N_e}{\alpha^*} = N_g\left[\frac{1-\alpha^*}{\alpha^*}\right] \qquad [3]$$

α^* is the value of α_g at equilibrium.

Supplementary Figures

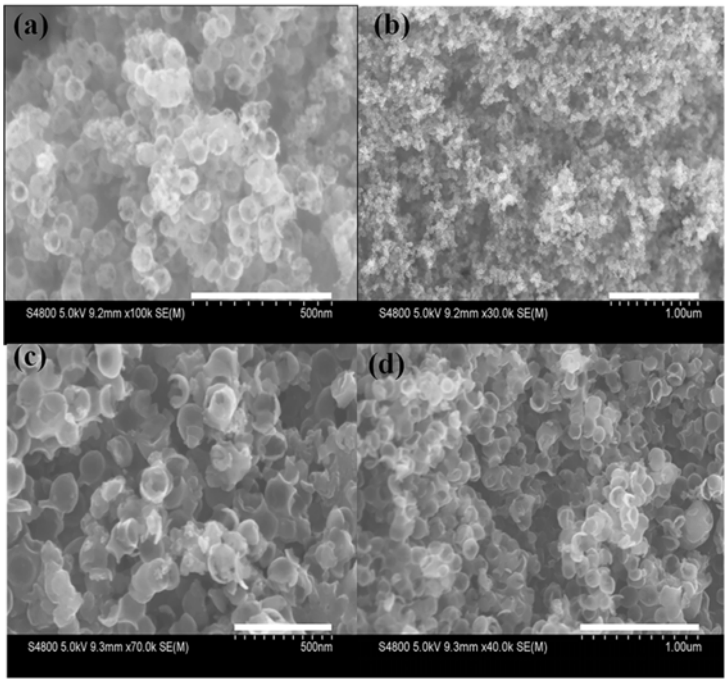

Supplementary Figure 1 SEM images: (a–b) N-C support; and (c–d) Co-N-C. Scale bar of (a, c) 500 nm and (b, d) 1µm.

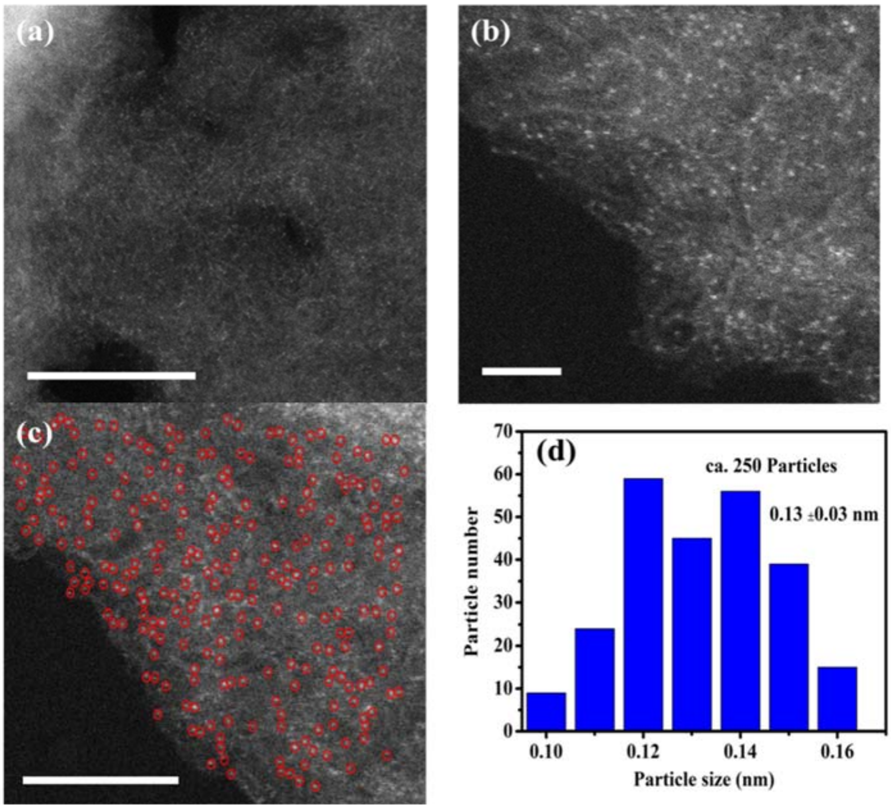

Supplementary Figure 2 (a–c) Three representative aberration-corrected high-angle annular dark field-scanning transmission electron microscopy images of fresh Co-N-C catalyst, all of them showing atomically dispersed Co atoms and (d) histogram of Co particle size distribution over Co-N-C. Scale bar: 10 nm of a, 2 nm of b and 5 nm of c.

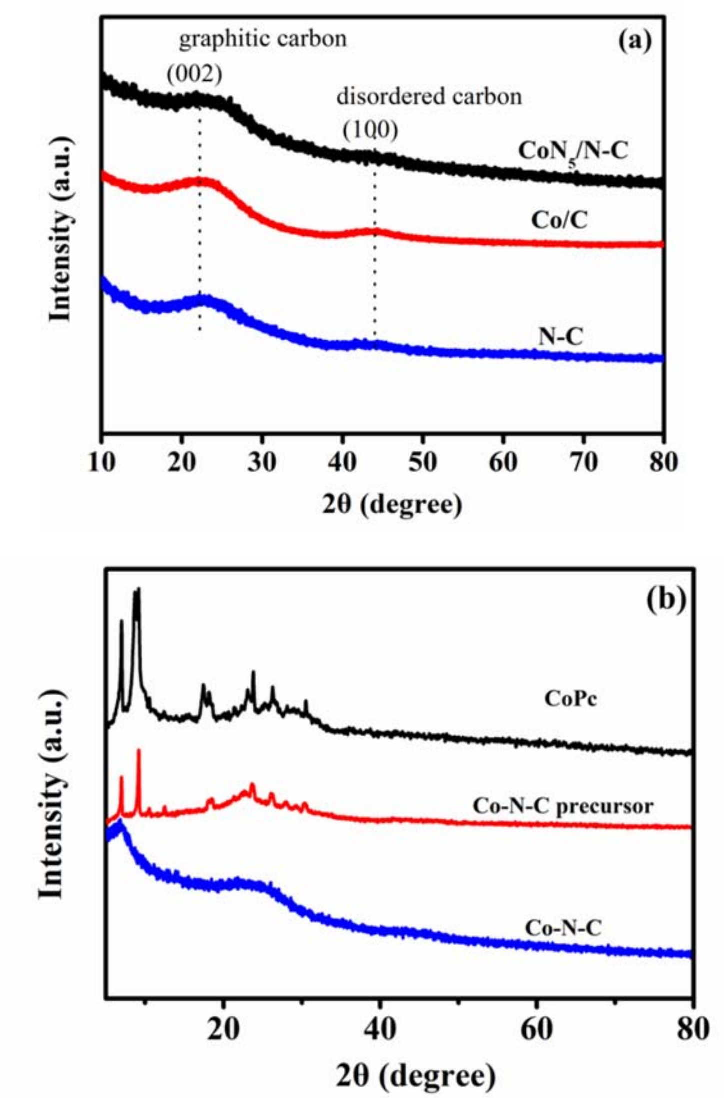

Supplementary Figure 3 (a) XRD patterns of N-C, Co/C and Co-N-C, and (b) comparison of XRD spectra of CoPc, Co-N-C precursor and Co-N-C. XRD patterns (Supplementary Figure 3a) also display no distinct differences between Co/C and N-C, with two peaks assignable to the (002) planes of graphitic and (100) planes of disordered carbon, respectively, which can also be verified by the D and G bands of Raman spectra (Supplementary Figure 4). Notably, no peaks related to metallic Co or other Co species are observed in the XRD pattern of Co/C, indicating high dispersity of the Co species anchored on the carbon support.

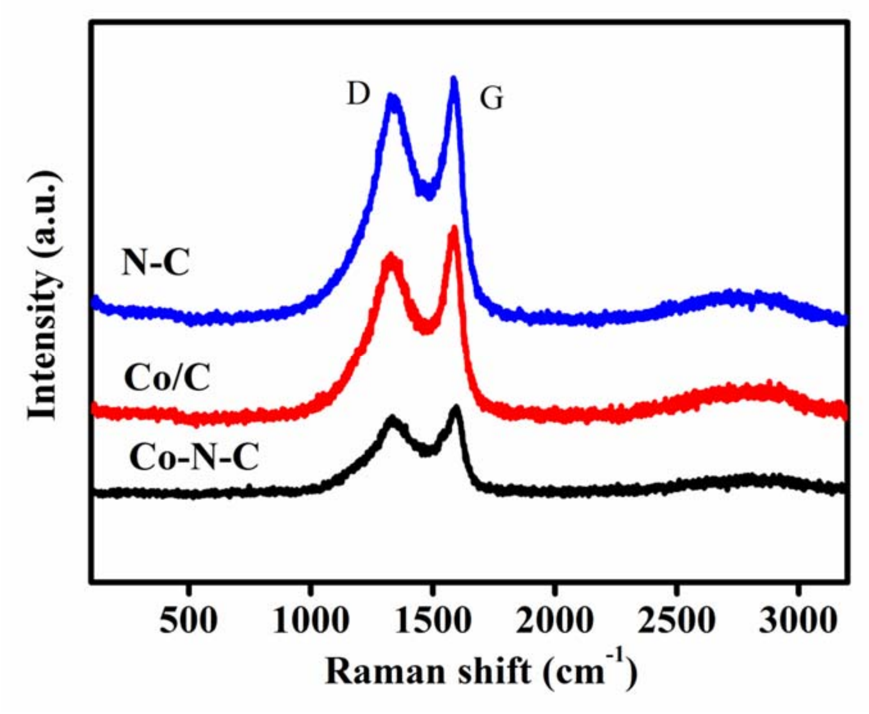

Supplementary Figure 4 Raman spectra of N-C, Co/C and Co-N-C.

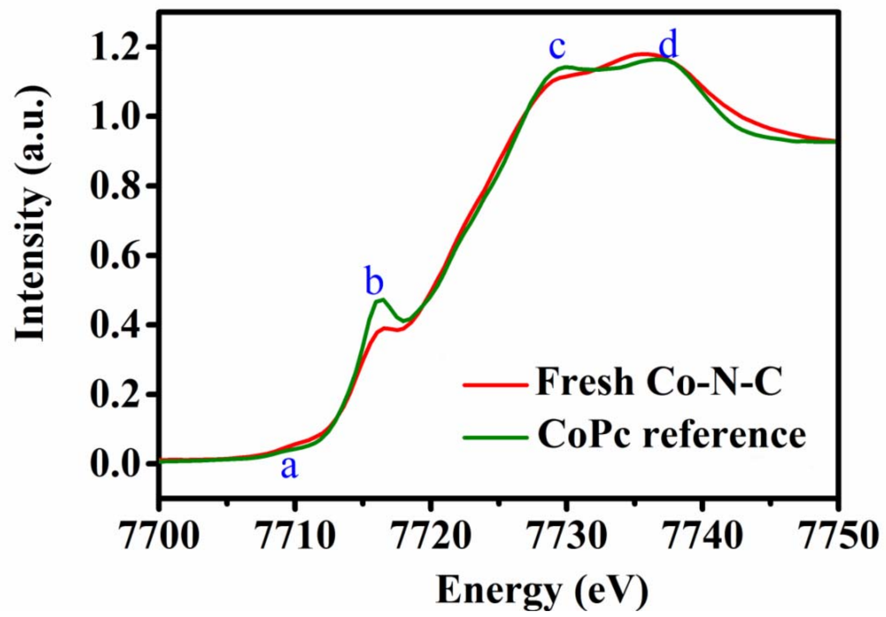

Supplementary Figure 5 Normalized Co K-edge XANES spectra of Co-N-C and CoPc reference. The Co-K edge XANES spectrum of CoPc (Supplementary Figure 5) exhibits several transitions (labeled a, b, c and d), and the energy values are 7709.5, 7716.5, 7716.5 and 7736.9 eV, respectively, corresponding to 1s→3d (p-d hybridization), 1s→4p_z + ligand hole, 1s→4p_{xy} + ligand hole, and 1s→4p_{xy} transitions. To be noted, the transition labeled b, which is observed for compounds with square-planar coordination is a fingerprint of the Co-N_4 structure and any modification of the environment would greatly affect this transition [3, 4]. Compared with the CoPc reference material, Co-N-C is obviously smaller in pre-edge peak intensity (feature b), indicating partial disruption of the planar central symmetry upon heat treatment.

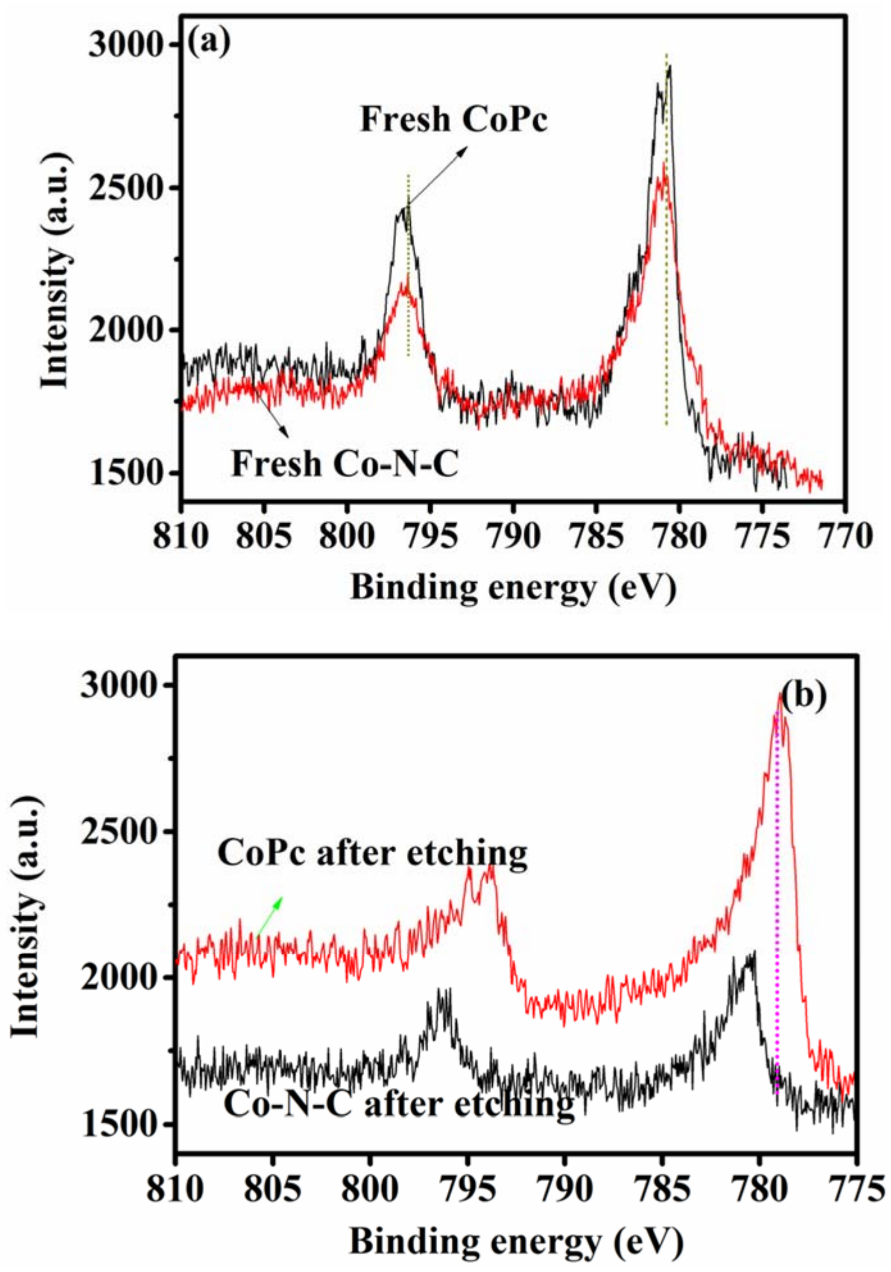

Supplementary Figure 6 XPS Co 2p spectra of (a) fresh Co-N-C and CoPc reference, and (b) Co-N-C and CoPc reference after Ar^+ etching.

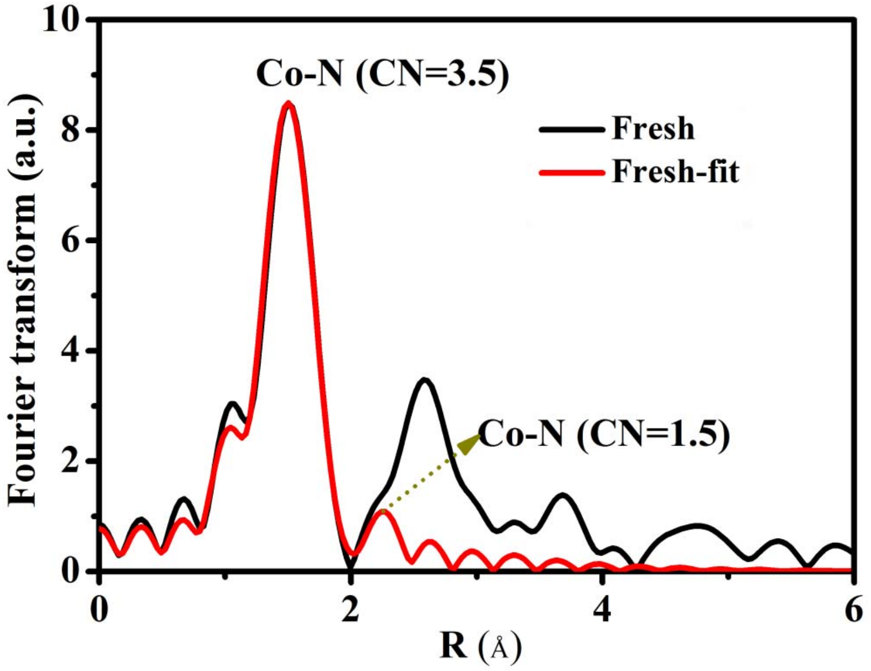

Supplementary Figure 7 Co K-edge EXAFS fitting curve of fresh Co-N-C catalyst.

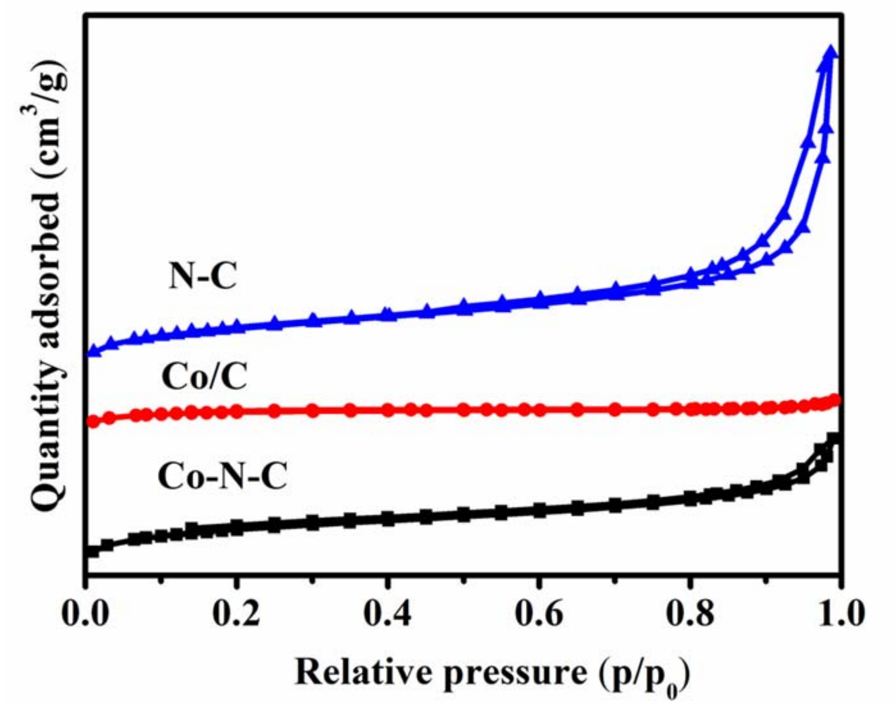

Supplementary Figure 8 N_2 adsorption-desorption isotherms of N-C, Co/C and Co-N-C. To be noted, N-C, Co-N-C and Co/C show a typical type-IV curve with hysteresis loops, suggesting the existence of mesopores.

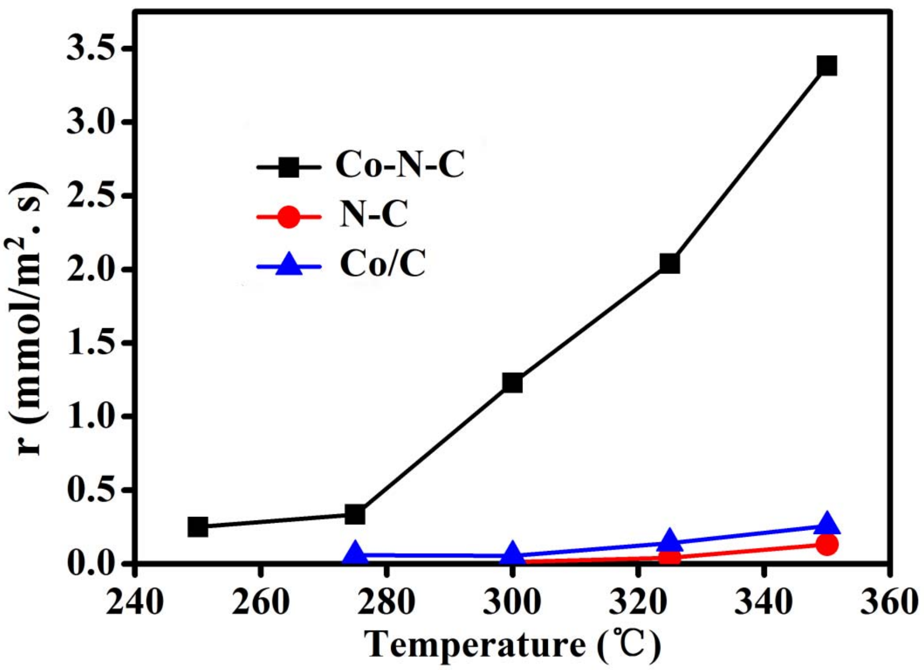

Supplementary Figure 9 Surface-area-normalized NH_3 synthesis rates of Co-N-C, Co/C and N-C at 1 MPa versus reaction temperatures.

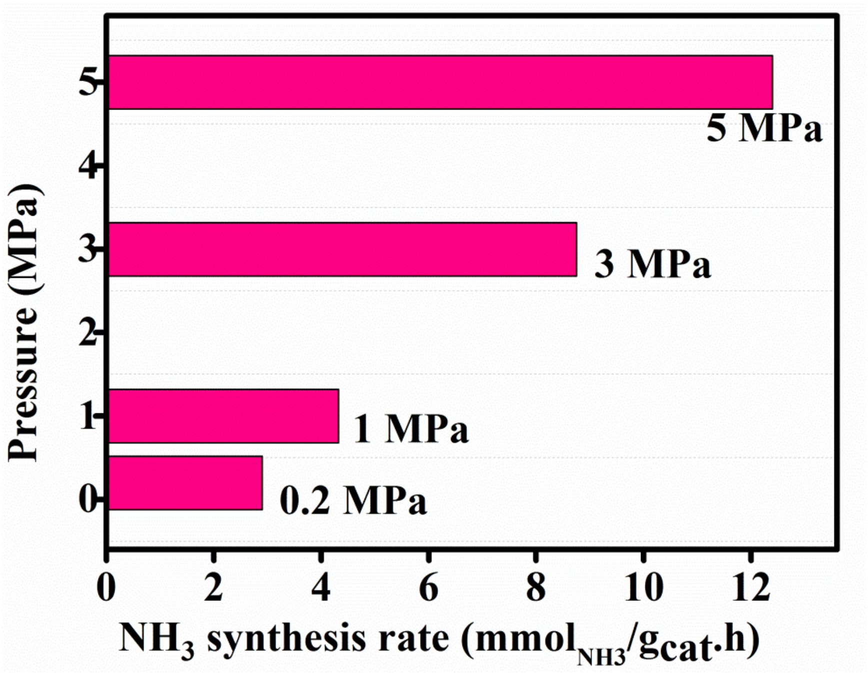

Supplementary Figure 10. NH_3 synthesis rates of Co-N-C versus pressures at 350 °C. For Co-N-C, it can be observed that NH_3 synthesis rate at 350 °C increases with increasing pressure, from 2.92 mol/(g_{cat}·h) at 0.2 MPa to 12.91 mol/(g_{cat}·h) at 5 MPa under 350 °C.

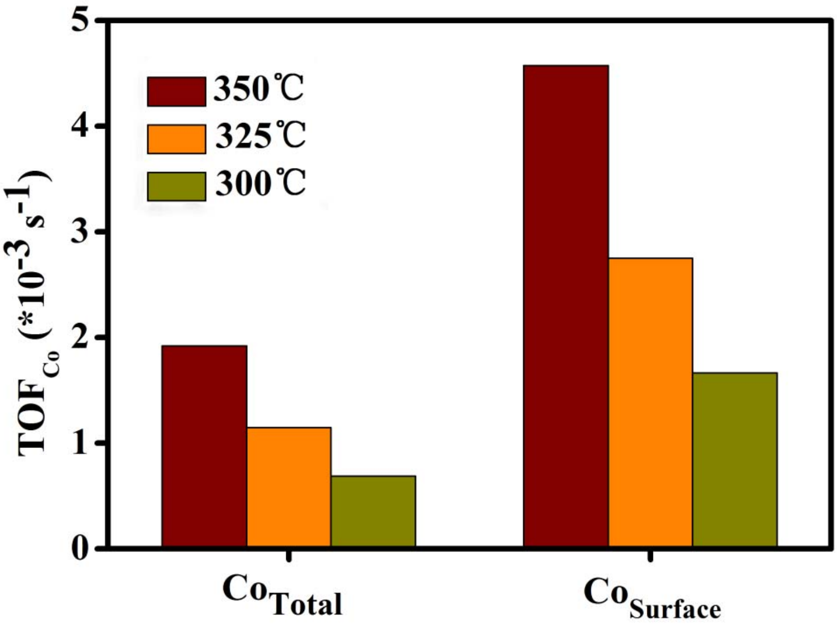

Supplementary Figure 11 Turnover frequencies (TOF_{Co}) of Co-N-C at 1 MPa and designated temperatures. In the present study, TOF_{Total} and $TOF_{surface}$ were acquired having the NH_3 synthesis rate divided by total number of Co atoms and surface number of Co atoms, respectively. On the basis of CO pulse experiment, we cannot accurately measure the number of Co active sites because the related Co dispersion data are not available.

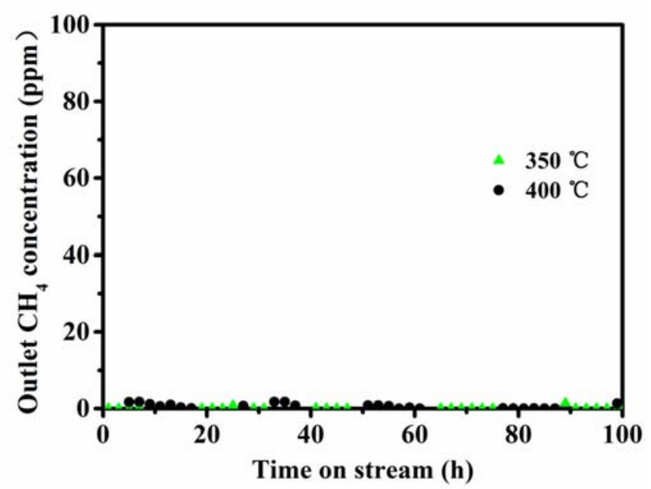

Supplementary Figure 12 The outlet CH_4 concentration as a function of time during NH_3 synthesis over Co-N-C sample at different temperatures (test conditions: WHSV= 60 000 ml · g $^{-1}$ · h $^{-1}$, 1MPa).

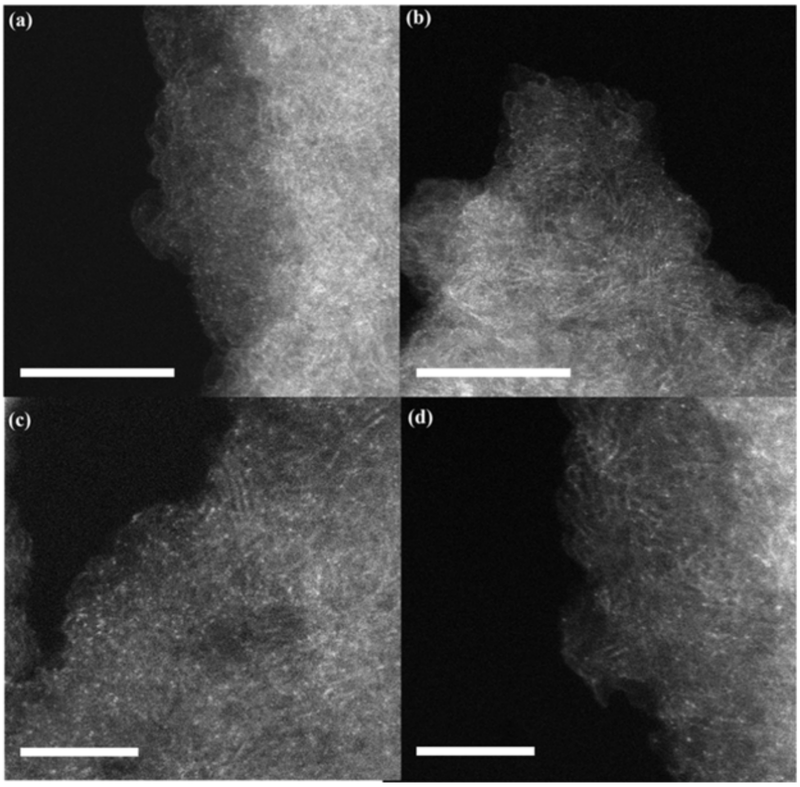

Supplementary Figure 13 Four representative aberration-corrected high-angle annular dark field-scanning transmission electron microscopy images of the Co-N-C catalyst after NH_3 synthesis stability test for 102 h at 350 ℃, showing the excellent stability of the atomically dispersed Co atoms. Scale bar of (a, b) 10 nm and (c, d) 5nm.

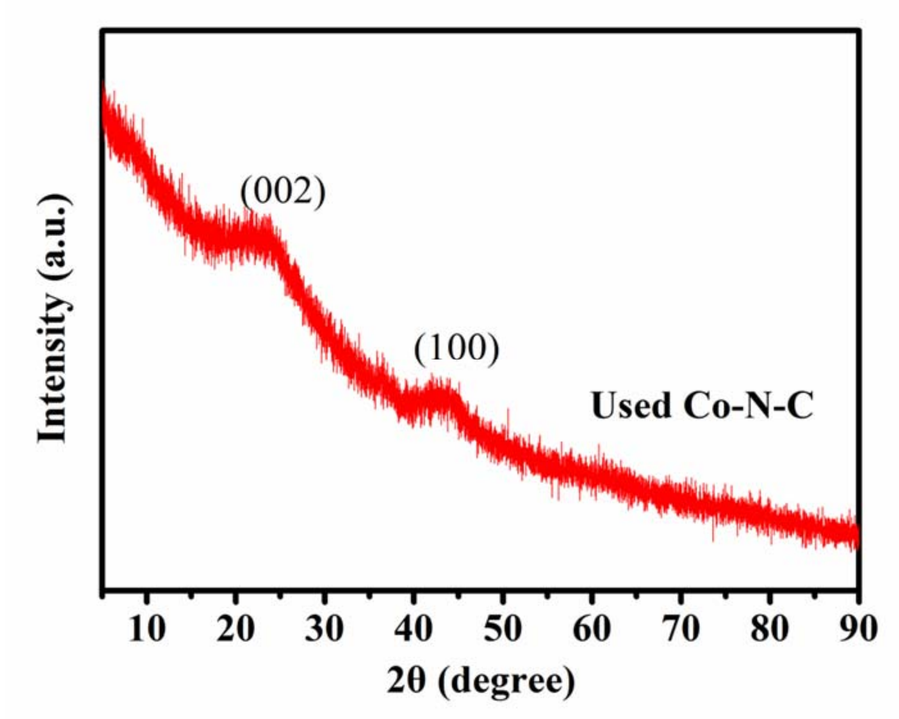

Supplementary Figure 14 XRD pattern of Co-N-C catalyst after NH_3 synthesis. There was no obvious change in phase property.

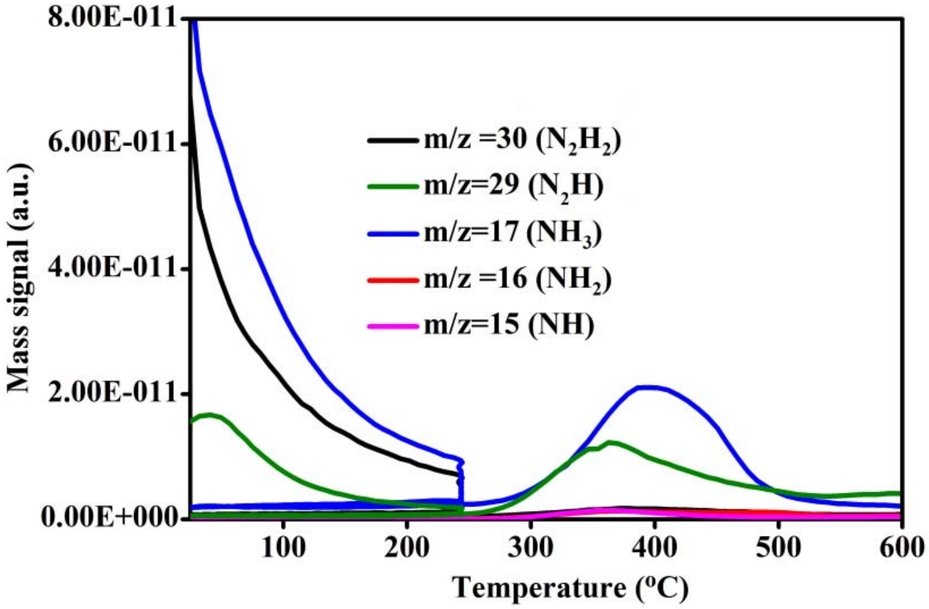

Supplementary Figure 15 Ar-TPD-MS profiles of Co-N-C after NH_3 synthesis at 1 MPa and 350 ℃ for 102 h. It can be observed that $*N_2H_2$ and NH_3 are the main desorption products, while NH (m/z=15) and NH_2 (m/z=16) signals can be ignored.

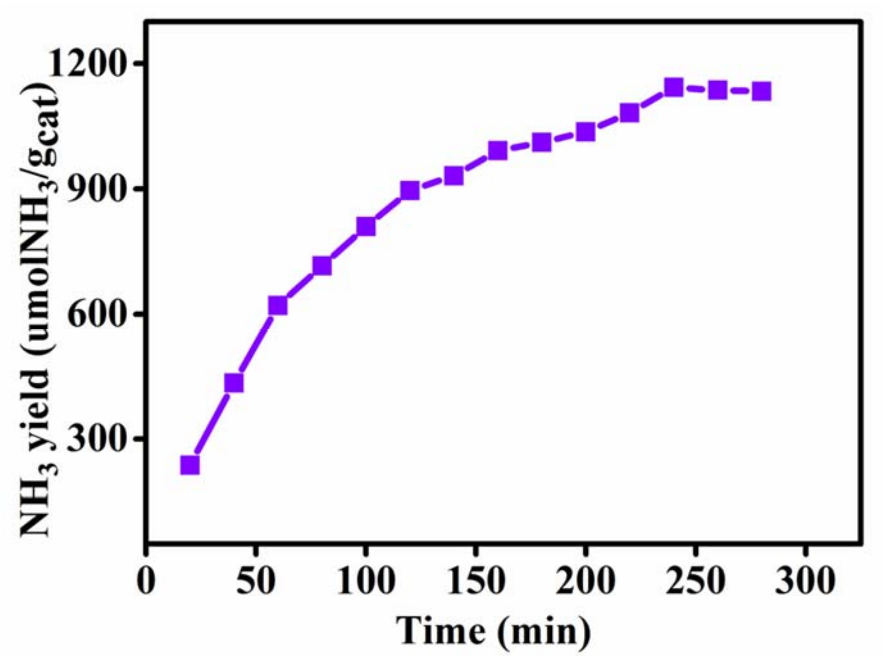

Supplementary Figure 16 NH_3 yield as a function of time over Co-N-C in the presence of 10%H_2/Ar at 350 ℃ and 1MPa.

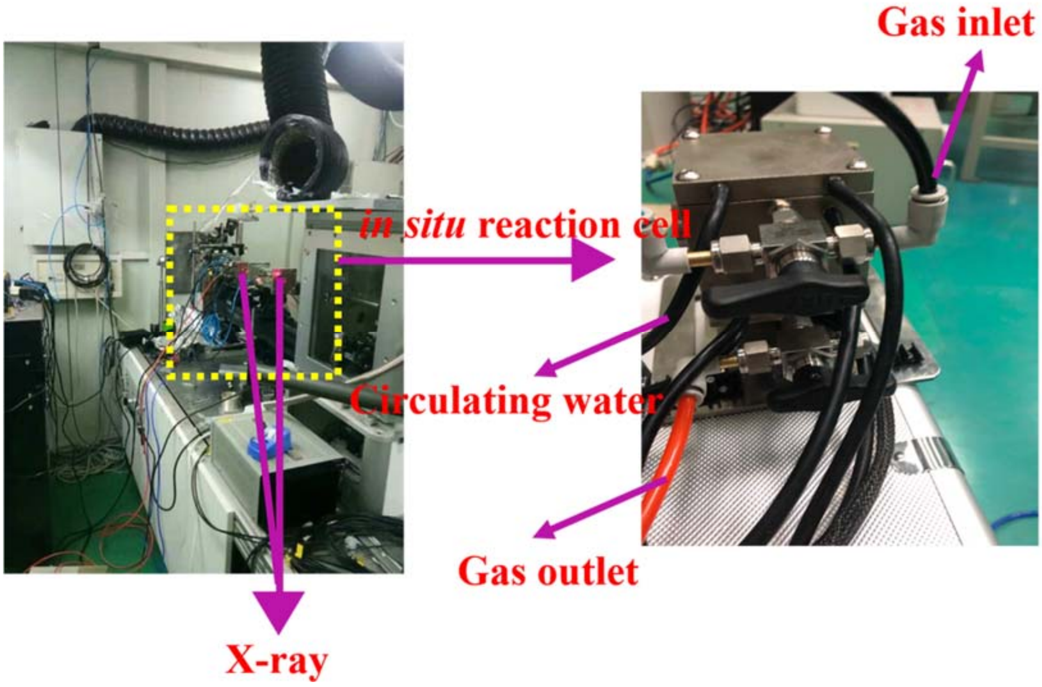

Supplementary Figure 17 Optical photograph of the equipment used for *in-situ* XAS characterization.

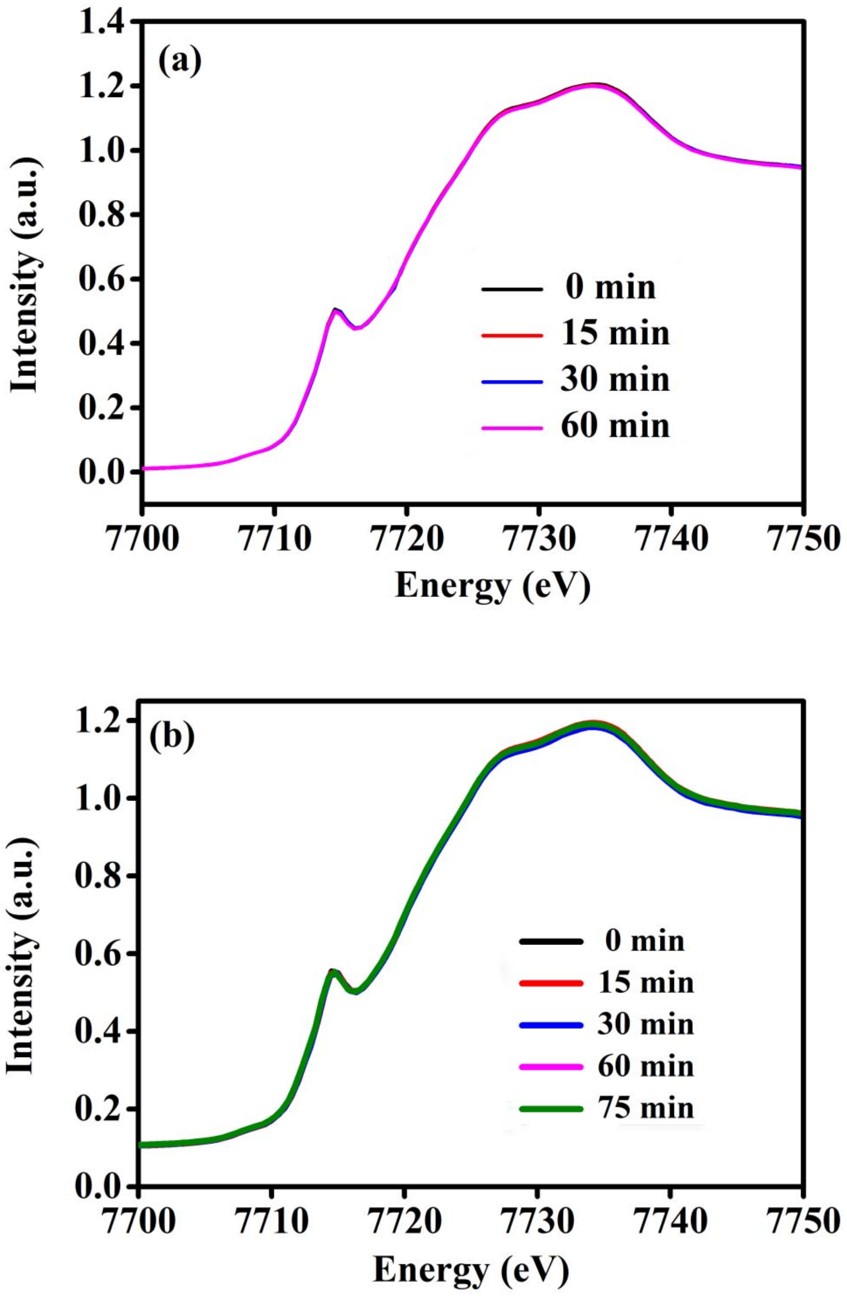

Supplementary Figure 18 *In-situ* XANES results of Co-N-C catalyst: (a) Co K-edge XANES spectra at 350 °C in the presence of 10%H_2/He as a function of time and (b) Co K-edge XANES spectra at 350 °C in the presence of N_2-H_2 mixture (V_{N2}:V_{H2}=1:3) as a function of time.

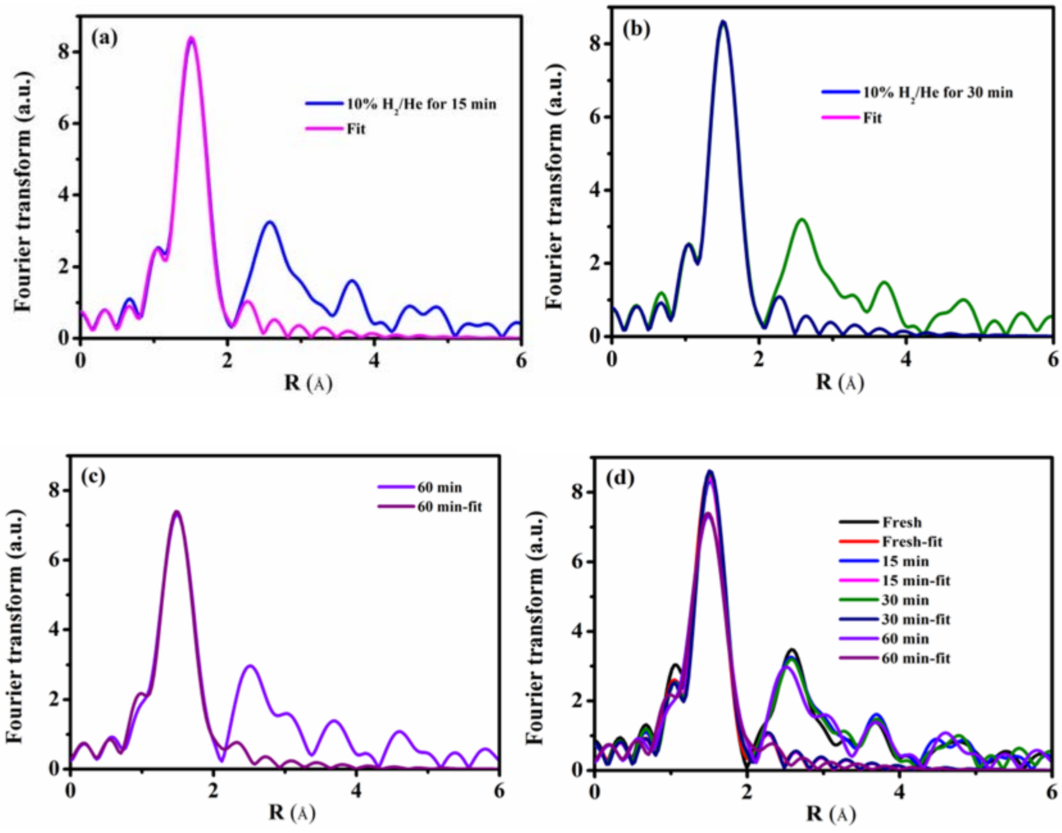

Supplementary Figure 19 (A-D) *In-situ* EXAFS results of Co-N-C catalyst: two shells fitting Co K-edge EXAFS curve of Co-N-C exposed at 350 °C in the presence of 10%H_2/He with different exposure times.

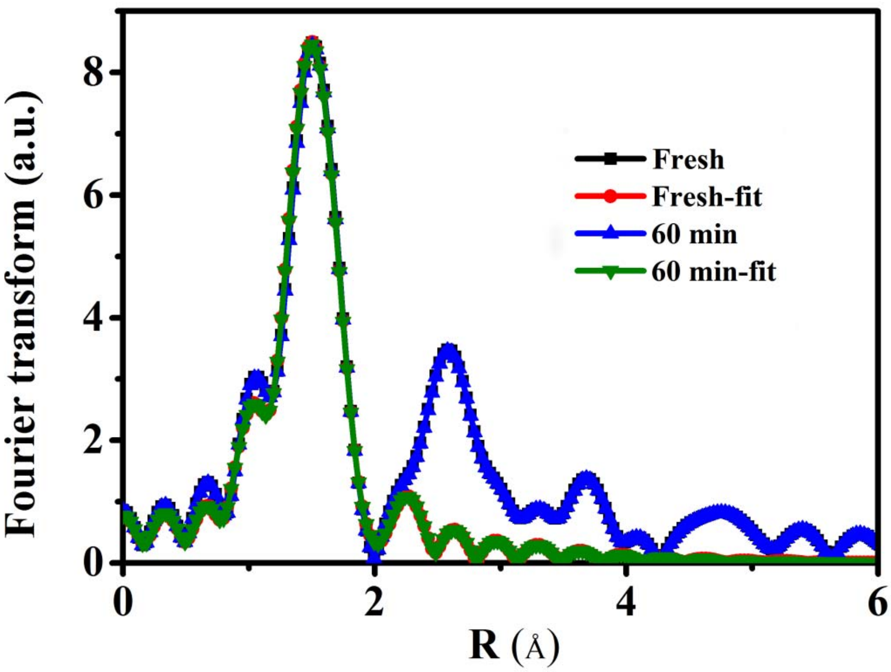

Supplementary Figure 20 *In-situ* EXAFS results of Co-N-C catalyst: two shells fitting Co K-edge EXAFS curve of Co-N-C exposed at 350 °C in the presence of N_2-H_2 mixture (V_{N2}:V_{H2}=1:3).

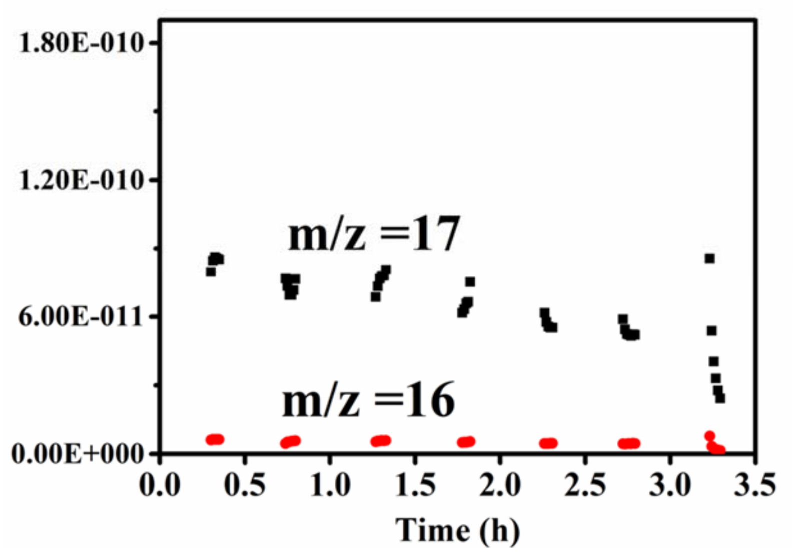

Supplementary Figure 21 The independent signals of m/z=17 and m/z=18 versus time from Figure 4c in the main text.

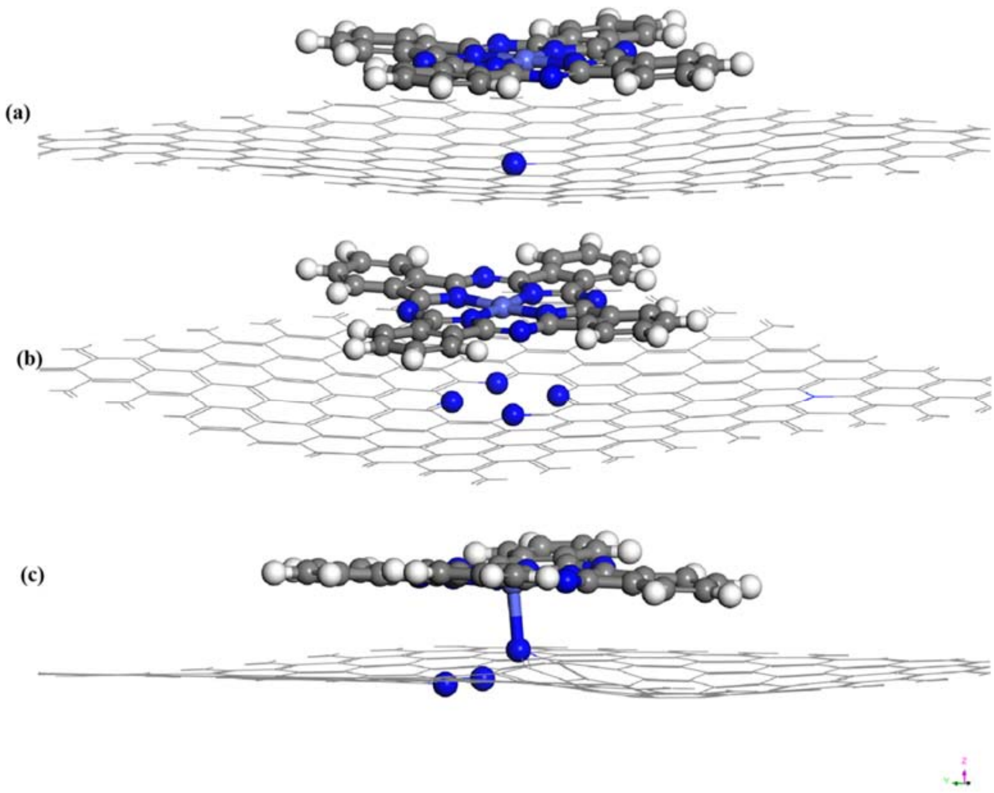

Supplementary Figure 22 Optimized configurations of (a) graphitic N, (b) pyrindinic N, and (c) pyrrolic N anchored with Co sites (N: blue; Co: blue grey).

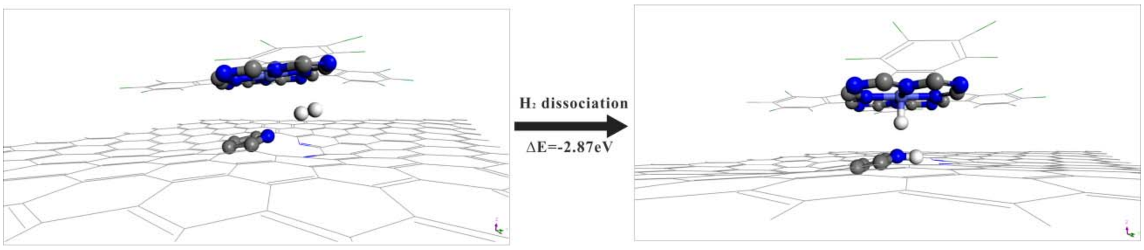

Supplementary Figure 23 H_2 dissociation on Co-N-C (black: C, white: H; N: blue; Co: blue grey).

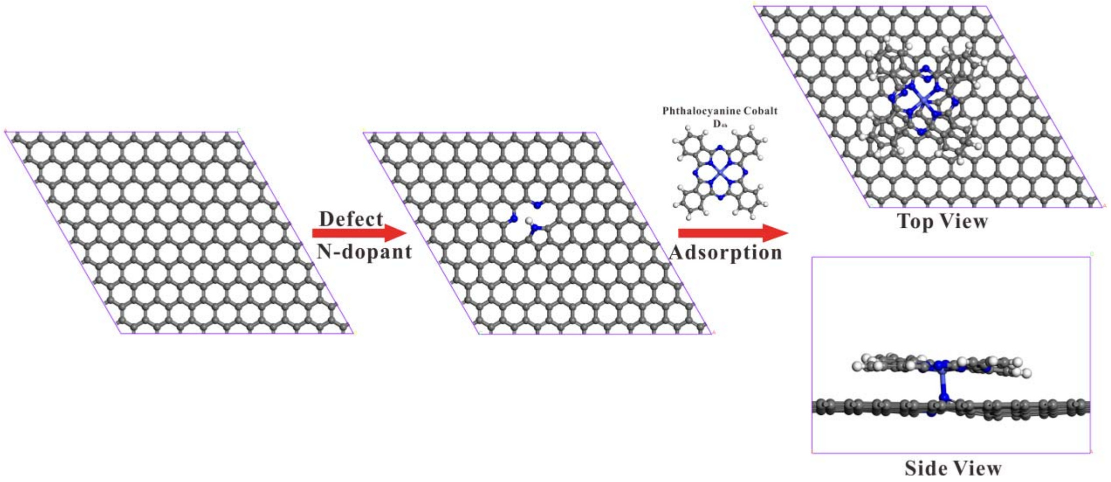

Supplementary Figure 24 Steps to construct model of Co-N-C for NH_3 synthesis reaction. First, we constructed a single layer graphene with 10*10*1 supercell, and then two connecting carbon atoms were defected to accommodate a nitrogen atom for the generation of pyrrolic N as site for coordination with the Co atom of phthalocyanine cobalt as shown in Supplementary Figure 24.

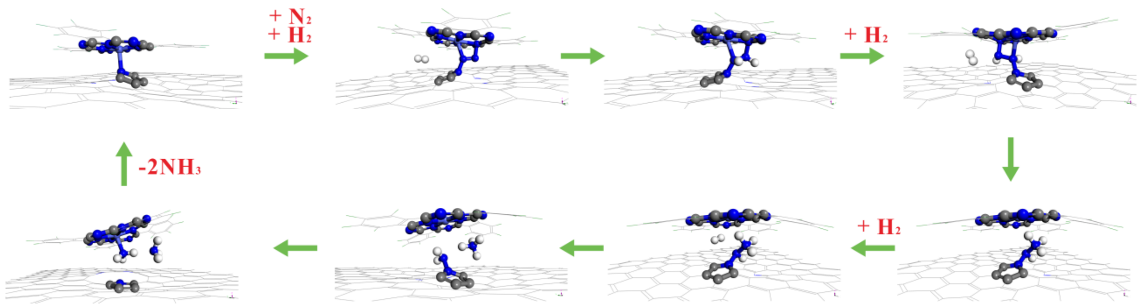

Supplementary Figure 25 Snapshots of the reaction $N_2 + 3H_2 \rightarrow 2NH_3$ on Co-N-C based on DFT calculation (black: C, white: H; N: blue; Co: blue grey).

Supplementary Tables

Supplementary Table 1 Textural properties, N and Co content, surface Co content, surface N/Co molar ratio and activation energy (E_a).

Sample	BET surface area (m^2/g)	Pore volume (cm^3/g)	Pore diameter (nm)	N content[a] (wt.%)	Co content[b] (wt.%)	Surface Co content[c] (wt.%)	Surface N/Co molar ratio[d]	E_a (kJ/mol)[e]
Co-N-C	356	0.23	7.64	5.35	3.73	1.57	6.10	50±7
Co/C	430	0.03	6.05	-	3.80	1.42	-	99±6
N-C	543	0.64	13.76	1.10	-		-	133±7

[a]Results based on elemental analysis;

[b]ICP results;

[c] Results based on *in situ* XPS under N_2-H_2 mixture atmosphere at 350 °C;

[d] Results based on XPS over fresh sample;

[e]The apparent activation energies (E_a) were measured on the basis of elimination of internal and external diffusion methods.[5, 6]

Supplementary Table 2 EXAFS fitting parameters at the Co K-edge for Co-N-C.

Sample	Shell	N[a]	R (Å)[b]	σ^2 ($Å^2 \cdot 10^{-3}$)[c]	ΔE_0 (eV)[d]	R factor (%)
Fresh	Co-N	3.5±0.7	1.89±0.02	3.3±1.0	5.5±3.0	0.6
	Co-N	1.5±0.3	2.35±0.03	4.8±1.0		
$10\%H_2/He$ for 15min	Co-N	3.4±0.5	1.91±0.01	2.0±0.5	5.4±1.5	0.2
	Co-N	1.0±0.2	2.35±0.02	5.7±1.0		
$10\%H_2/He$ for 30 min	Co-N	3.2±0.3	1.91±0.01	2.0±0.5	5.4±1.5	0.2
	Co-N	0.9±0.2	2.35±0.02	5.7±1.0		
$10\%H_2/He$ for 60 min	Co-N	3.5±0.4	1.90±0.01	3.4±1.0	4.2±1.8	0.6
	Co-N	0.9±0.2	2.35±0.02	4.1±0.9		
N_2-H_2 mixture for 60 min	Co-N	3.5±0.7	1.90±0.02	2.5±1.4	5.4±3.0	0.6
	Co-N	1.5±0.3	2.35±0.03	5.0±1.0		
Co foil	Co-Co	12*	2.50±0.01	6.4±0.1	6.6±0.3	0.1
CoPc	Co-N	4.6±1.0	1.91±0.01	2.5±1.4	9.1±3.5	0.1

[a]*CN*: coordination numbers;

[b]*R*: bond distance;

[c]σ^2: Debye-Waller factors;

[d]ΔE_0: the inner potential correction.

R factor: goodness of fit.

S_0^2, 0.8, was obtained from the experimental EXAFS fit of CoPc reference by fixing CN as the known crystallographic value and was fixed to all the samples.

Supplementary Table 3 NH_3 synthesis performance over various Co-based catalysts.

Sample	Co loading (wt.%)	Reaction Conditions			NH_3 synthesis rate ($mmol_{NH3}/g_{cat}\cdot h$)	NH_3 synthesis rate ($mmol_{NH3}/g_{Co}\cdot h$)	TOF_{Co} ($10^{-3}s^{-1}$)[a]	E_a (kJ/mol)	Ref.
		T (℃)	P (MPa)	WHSV (60 000 ml · $g^{-1}\cdot h^{-1}$)					
Co-N-C	3.73	350	1.0	60 000	4.34	116.35	1.91	50±7	This work
Co/C12A7:e^-	2.60	340	0.1	18 000	0.912	35.08	0.574	49.5	[7]
BaH_2-Co/CNTs	5.20	300	1.0	60 000	4.80	92.31	1.51	58	[8]
BaO-Co/CNTs	3.25	300	1.0	60 000	0.029	0.89	0.015	136	[8]
LaCoSi	26.1	400	0.1	36 000	1.25	4.78	0.078	42	[9]
Co-LiH	59.8	350	1.0	60 000	12.0	20.07	0.33	52	[10]
Co_3Mo_3N	35.2	500	0.1	12 000	0.49	1.39	-	-	[11]
Cs−Co_3Mo_3 N	36.9	400	0.1	9 000	0.986	2.67	-	57	[12]
$Ba_{0.35}$–Co/C	-	400	1.0	53 400	$6.2*10^{-3}$	-	-	102	[13]
$Sr_{0.35}$–Co/C	-	400	1.0	53 400	$1.4*10^{-3}$	-	-	110	[13]

[a]This value was calculated from the rate of NH_3 synthesis divided by the number of total Co metal atoms.

Supplementary Table 4 XPS data of various surface N species information in the case of Co-N-C.

Co-N-C	Graphitic N / Total surface N (%)	Pyrrolic N / Total surface N (%)	Pyridinic N / Total surface N (%)	Oxidized N / Total surface N (%)
Fresh	19.5	24.6	38.9	17.0
10%H_2/He	22.1	35.7	24.4	17.8
25%N_2-75%H_2	24.9	22.5	38.7	13.9

Supplementary Table 5 DFT calculation results of Co-N and H-H bond lengths and binding energy in the case of Co-N-C.

Co-N-C	d_{Co-N}[a] (Å)	d_{H-H}[b] (Å)	E_{ads}[c] (eV)
Graphitic N	3.45	0.77	0.84
Pyrrolic N	2.14	0.75	0.43
Pyridinic N	3.36	2.31	-1.98

[a]d_{Co-N} represents the bond lengths of Co-N after Co species interaction with various N species.

[b]d_{H-H} represents the bond lengths of H-H species after H_2 adsorption on the N species that interacted with Co.

[c]E_{ads} referes to the binding energies after H_2 adsorption on the N species that interacted with Co.

Supplementary References

1. Hunter, S. M., Gregory, D. H., Hargreaves, J. S. J., Richard, M., Duprez, D. & Bion, N. A Study of $^{15}N/^{14}N$ Isotopic Exchange over Cobalt Molybdenum Nitrides. *ACS Catal.* **3**, 1719–1725, (2013).

2. Duprez, D., Hargreaves, J. S. J., Jackson, S. D. & Webb, G. Eds. In Isotopes in Heterogeneous Catalysis. *Imperial College Press: London.* **6**, 133, (2006).

3. Martins Alves, M. C., Dodelet, J. P., Guay, D., Ladouceur, M. & Tourillon, Origin of the Electrocatalytic Properties for O_2 Reduction of Some Heat-treated Polyacrylonitrile and Phthalocyanlne Cobalt Compounds Adsorbed on Carbon Black as Probed by Electrochemistry and X-ray Absorption Spectroscopy, *J. Phys. Chem*. **96**, 10898-10905, (1992).

4. G.Liu,W., &Zhang, T., et. al.Single-atom Dispersed Co-N-C Catalyst: Structure

Identification and Performance for Hydrogenative Coupling of Nitroarenes. *Chem. Sci.,* **7**, 5758-5764, (2016).

5. Lin, B., Heng, L., Fang, B., Yin, H., Ni, J., Wang, X., Lin, J. & Jiang. L. Ammonia Synthesis Activity of Alumina-supported Ruthenium Catalyst Enhanced by Alumina Phase Transformation. *ACS Catal*. **9**, 1635–1644, (2019).

6. Aiki, K., Kumasaka, M., Oma, T., Kato, O., Matsuda, H., Watsuda, N., Watanabe, N., Yamazki, K., Ozaki, A. & Onishi, T. Support and Promoter Effect of Ruthenium Catalyst III: Kinetic of Ammonia Synthesis over Various Ru Catalysts. *Appl. Catal. A: Gen.* **28**, 51–68, (1986).

7. Inoue, Y., Kitano, M., Tokunari, M., Taniguchi, T., Ooya, K., Abe, H., Niwa, Y., Sasase, M., Hara, M. & Hosono, H. Direct Activation of Cobalt Catalyst by $12CaO \cdot 7Al_2O_3$ Electride for Ammonia Synthesis. *ACS Catal.* **9**, 1670–1679, (2019).

8. Gao, W., Wang, P., Guo, J., Chang, F., He, T., Wang, Q., Wu, G. & Chen, P. Barium Hydride-mediated Nitrogen Transfer and Hydrogenation for Ammonia Synthesis: A Case Study of Cobalt. *ACS Catal.* **7**, 3654–3661, (2017).

9. Gong, Y., Wu, J., Kitano, M., Wang, J., Ye, T-N., Li, J., Kobayashi, Y., Kishida, K., Abe, H., Niwa, Y., Yang, H., Tada, T. & Hosono, H. Ternary Intermetallic LaCoSi as a Catalyst for N_2 Activation, *Nat. Catal.* **1**, 178–185, (2018).

10. Wang, P. K., Chang, F., Gao, W. B., Guo, J. P., Wu, G. T., He, T. & Chen, P. Breaking Scaling Relations to Achieve Low-temperature Ammonia Synthesis through LiH-Mediated Nitrogen Transfer and Hydrogenation. *Nat. Chem.* **9**, 64–70, (2017).

11. AlShibane, I., Daisley, A., Hargreaves, J. S. J., Hector, A. L., Laassiri, S., Rico, J. L., & Smit, R. I. The Role of Composition for Cobalt Molybdenum Carbide in Ammonia synthesis. *ACS Sustain. Chem. Eng.* **5**, 9214–9222, (2017).

12. Kojima, R., Aika, K. Cobalt Molybdenum Bimetallic Nitride Catalysts for Ammonia Synthesis Part 2. Kinetic Study. *Appl. Catal. A.* **218**, 121–128, (2001).

13. Hagen, S., Barfod, R., Fehrmann, R., Jacobsen, C. J. H., Teunissen, H. T. & Chorkendorff, I. Ammonia Synthesis with Barium-promoted Iron-cobalt Alloys Supported on Carbon. *J. Catal.* **214**, 327–335, (2003).
